# Supplementary material for: The nitrogen removal characterization and ecological risk assessment of Bacillus sp. isolated from mariculture systems in China with spatiotemporal difference
Source: PLoS One. 2025 Mar 20;20(3):e0319344. doi: 10.1371/journal.pone.0319344 (PMC11925278; doi:10.1371/journal.pone.0319344)
Supplement: S1 Table — (DOC) [file pone.0319344.s001.doc]

| **Medium** | **Components** |
| --- | --- |
| NDM | NH_4_Cl 2 g, C_6_H_12_O_6_·H_2_O 5g, NaCl 2 g, K_2_HPO_4_ 1 g, MgSO_4_·7H_2_O 0.5 g, H_2_O 1L, agar 15 g, pH 7.2-7.4. |
| HNM | NH_4_Cl 0.5 g, C_6_H_12_O_6_·H_2_O 5g, K_2_HPO_4_ 0.5 g, sterilized seawater 1 L, agar 18 g, pH 7.0. |
| HDM | NaNO_2_ 0.2 g, C_6_H_12_O_6_·H_2_O 5 g, K_2_HPO_4_ 0.5 g, sterilized seawater 1 L, agar 18 g, pH 7.0. |
| DM-1 | KNO_3_ 0.2 g, C_6_H_12_O_6_·H_2_O 5g, K_2_HPO_4_ 0.5 g, sterilized seawater 1 L, agar 18 g, pH 7.0. |
| BTBM | L-Asparagine 10 g, KNO_3_ 1 g, KH_2_PO_4_ 1 g, FeCl_2_·6H_2_O 0.05 g, CaCl_2_·2H_2_O 0.2 g, MgSO_4_·7H_2_O 1 g, 1% bromothymol blue 1 mL, agar 20 g, H_2_O 1 L, pH 7.0. |
| DM-2 | KNO_3_ 0.5 g, CH_3_COONa 2.0 g, MgSO_4_·7H_2_O 0.6 g, CaCl_2_·2H_2_O 0.07 g, KH_2_PO_4_ 0.4 g, Tris buffer 12 mL, pH 7.0, trace element solution 1 mL. The trace element solution per liter consists of: ZnSO_4_ 2.2 g, FeSO_4_·7H_2_O 3.0 g, CaCl_2_ 5.5 g, MnCl_2_·4H_2_O 5 g, CuSO_4_·5H_2_O 1.6 g, CoCl_2_·6H_2_O 1.6 g, (NH_4_)_6_Mo_7_O_4_·2H_2_O 1.1 g. |
| INDTM | C_6_H_12_O_6_·H_2_O 2.0808 g, NH_4_Cl 0.0535 g, NaNO_2_ 0.069 g, NaNO_3_ 0.085 g, seawater 1 L, pH7.5 |
