# Supplementary material for: The nitrogen removal characterization and ecological risk assessment of Bacillus sp. isolated from mariculture systems in China with spatiotemporal difference
Source: PLoS One. 2025 Mar 20;20(3):e0319344. doi: 10.1371/journal.pone.0319344 (PMC11925278; doi:10.1371/journal.pone.0319344)
Supplement: S2 Table — (DOC) [file pone.0319344.s002.doc]

| **Strain number** | **Strain name** | **Time** | **Location** | **Conditions** | **Strain number** | **Strain name** | **Time** | **Location** | **Conditions** |
| --- | --- | --- | --- | --- | --- | --- | --- | --- | --- |
| **B1** | *B. velezensis* | 2021 | Jiangsu | shimp | **B29** | *B. velezensis* | 2020 | Shandong | shimp culture pond water |
| **B2** | *B. velezensis* | 2021 | Guangdong | shimp | **B30** | *B. velezensis* | 2018 | Shangdong | squid culture pond water |
| **B3** | *B. velezensis* | 2020 | Hebei | shimp | **B31** | *B. stercoris* | 2021 | Hebei | shimp |
| **B4** | *B. velezensis* | 2020 | Hebei | shimp | **B32** | *B. nealsonii* | 2020 | Shangdong | shimp |
| **B5** | *B. velezensis* | 2021 | Guangdong | bait | **B33** | *B. nealsonii* | 2021 | Guangdong | shimp culture pond water |
| **B6** | *B. stercoris* | 2021 | Guangdong | shrimp culture pond sediment | **B34** | *B. stratosphericus* | 2021 | Guangdong | shimp |
| **B7** | *B. stercoris* | 2021 | Guangdong | shimp culture pond water | **B35** | *B. flexus* | 2020 | Hebei | shimp |
| **B8** | *B. stercoris* | 2020 | Hebei | shimp | **B36** | *B. flexus* | 2016 | Shangdong | grouper |
| **B9** | *B. inaquosorum* | 2020 | Hebei | shimp | **B37** | *B. flexus* | 2020 | Hebei | shimp |
| **B10** | *B. spizizenii* | 2021 | Guangdong | shrimp culture pond sediment | **B38** | *B. megaterium* | 2017 | Shangdong | sea cucumber |
| **B11** | *B. subtilis* | 2010 | Hainan | shimp | **B39** | *B. megaterium* | 2021 | Hainan | shimp |
| **B12** | *B. subtilis* | 2020 | Hebei | shimp | **B40** | *B. subtilis* | 2017 | Shangdong | sea cucumber |
| **B13** | *B. subtilis* | 2020 | Hebei | shimp | **B41** | *B. aryabhattai* | 2021 | Guangdong | shimp |
| **B14** | *B. subtilis* | 2020 | Hebei | shimp | **B42** | *B. aryabhattai* | 2021 | Guangdong | shimp |
| **B15** | *B. subtilis* | 2020 | Hebei | shimp | **B43** | *B. lehensis* | 2016 | Hainan | grouper |
| **B16** | *B. subtilis* | 2020 | Hebei | shimp | **B44** | *B. gibsonii* | 2021 | Guangdong | shimp |
| **B17** | *B. subtilis* | 2021 | Guangdong | shimp | **B45** | *B. methylotrophicus* | 2017 | Sahndong | sea cucumber |
| **B18** | *B. subtilis* | 2021 | Guangdong | bait | **B46** | *B. licheniformis* | 2021 | Guangdong | shimp |
| **B19** | *B. subtilis* | 2021 | Jiangsu | shimp culture pond water | **B47** | *B. altitudinis* | 2020 | Hebei | shimp |
| **B20** | *B. subtilis* | 2021 | Jiangsu | shimp culture pond water | **B48** | *B. haikouensis* | 2021 | Jiangsu | shrimp culture pond sediment |
| **B21** | *B. subtilis* | 2020 | Hebei | shimp | **B49** | *B. circulans* | 2021 | Guangdong | shimp |
| **B22** | *B. subtilis* | 2021 | Jiangsu | shimp | **B50** | *B. marisflavi* | 2020 | Hebei | shimp |
| **B23** | *B. subtilis* | 2021 | Jiangsu | shimp | **B51** | *B. subtilis* | 2020 | Hebei | shimp |
| **B24** | *B. subtilis* | 2021 | Jiangsu | shimp | **B52** | *B. subtilis* | 2020 | Hebei | shimp |
| **B25** | *B. subtilis* | 2021 | Jiangsu | shimp | **B53** | *B. subtilis* | 2021 | Jiangsu | shrimp culture pond sediment |
| **B26** | *B. subtilis* | 2020 | Hebei | shimp | **B54** | *B. subtilis* | 2021 | Guangdong | shrimp culture pond sediment |
| **B27** | *B. subtilis* | 2021 | Guangdong | shrimp culture pond sediment | **B55** | *B. subtilis* | 2021 | Guangdong | shimp |
| **B28** | *B. velezensis* | 2021 | Guangdong | shimp culture pond water |  |  |  |  |  |
