# Supplementary material for: The nitrogen removal characterization and ecological risk assessment of Bacillus sp. isolated from mariculture systems in China with spatiotemporal difference
Source: PLoS One. 2025 Mar 20;20(3):e0319344. doi: 10.1371/journal.pone.0319344 (PMC11925278; doi:10.1371/journal.pone.0319344)
Supplement: S3 Table — (DOC) [file pone.0319344.s003.doc]

| **Gene** | **Primer sequence（5′-3′）** | **Amplicon size (bp)** | **Reference** |
| --- | --- | --- | --- |
| *tentA* | F: GCTACATCCTGCTTGCCTTC | 212 | [22] |
|  | R: GCATAGATCGCCGTGAAGAG |  |  |
| *tentB* | F: TACGTGAATTTATTGCTTCGG | 206 |  |
|  | R: ATACAGCATCCAAAGCGCAC |  |  |
| *blaTEM* | F: AAAGATGCTGAAGATCA | 425 | [23] |
|  | R: TTTGGTATGGCTTCATTC |  |  |
| *ampC* | F: GCGAAAGCCAGCTGTCGGGC | 550 | [24] |
|  | R: CCYTTTTATGTACCCAYGA |  |  |
| *ant*(3’)*-Ia*(*aadA*) | F: ATCTGGCTATCTTGCTGACA | 388 | [25] |
|  | R: TTGGTGATCTCGCCTTTC |  |  |
| *aph*(6’)-*Id*(*strB*) | F: ATGTTCATGCCGCCTGTTTTT | 837 |  |
|  | R: CTAGTATGACGTCTGTCGC |  |  |
| *ermA* | F: GTTCAAGAACAATCAATACAGAG | 421 | [26] |
|  | R: GGATCAGGAAAAGGACATTTTAC |  |  |
| *ermX* | F: GTTGCGCTCTAACCGCTAAGGC | 566 |  |
|  | R: CCATGGGGACCACTGAGCCGTC |  |  |
| *floR* | F: CTGCTGATGGCTCCTTTC | 650 | [27] |
|  | R: GCCGTGGCGTAACAGAT |  |  |
| *qnrA* | F: TTCAGCAAGAGGATTTCTCA | 500 |  |
|  | F: GGCAGCACTATTACTCCCAA |  |  |
| *qnrB* | F: CCTGAGCGGCACTGAATTTAT | 617 |  |
|  | R: GTTTGCTGCTCGCCAGTCGA |  |  |
| *cfr* | F: TGAAGTATAAAGCAGGTTGGGAGTCA | 746 | [28] |
|  | R: ACCATATAATTGACCACAAGCAGC |  |  |
| *nor* | F: GGGCGCTGCGATGAAATTTAT | 1917 | [29] |
|  | R: GATAAAGGGCTATCCTATGC |  |  |
| *nap* | F: GGGCCATGAAATCTTTTGCCACAC | 2040 |  |
|  | R: TCATGCTTTTTCAATCTGTACACGTCC |  |  |
| *narG* | F: ATCAGCCACGCATCAGGCTC | 256 | This study |
|  | R: AAAATGCGCGTCAGGCGTTC |  |  |
