# Supplementary figures and images for: The nitrogen removal characterization and ecological risk assessment of Bacillus sp. isolated from mariculture systems in China with spatiotemporal difference
Source: PLoS One. 2025 Mar 20;20(3):e0319344. doi: 10.1371/journal.pone.0319344 (PMC11925278; doi:10.1371/journal.pone.0319344)

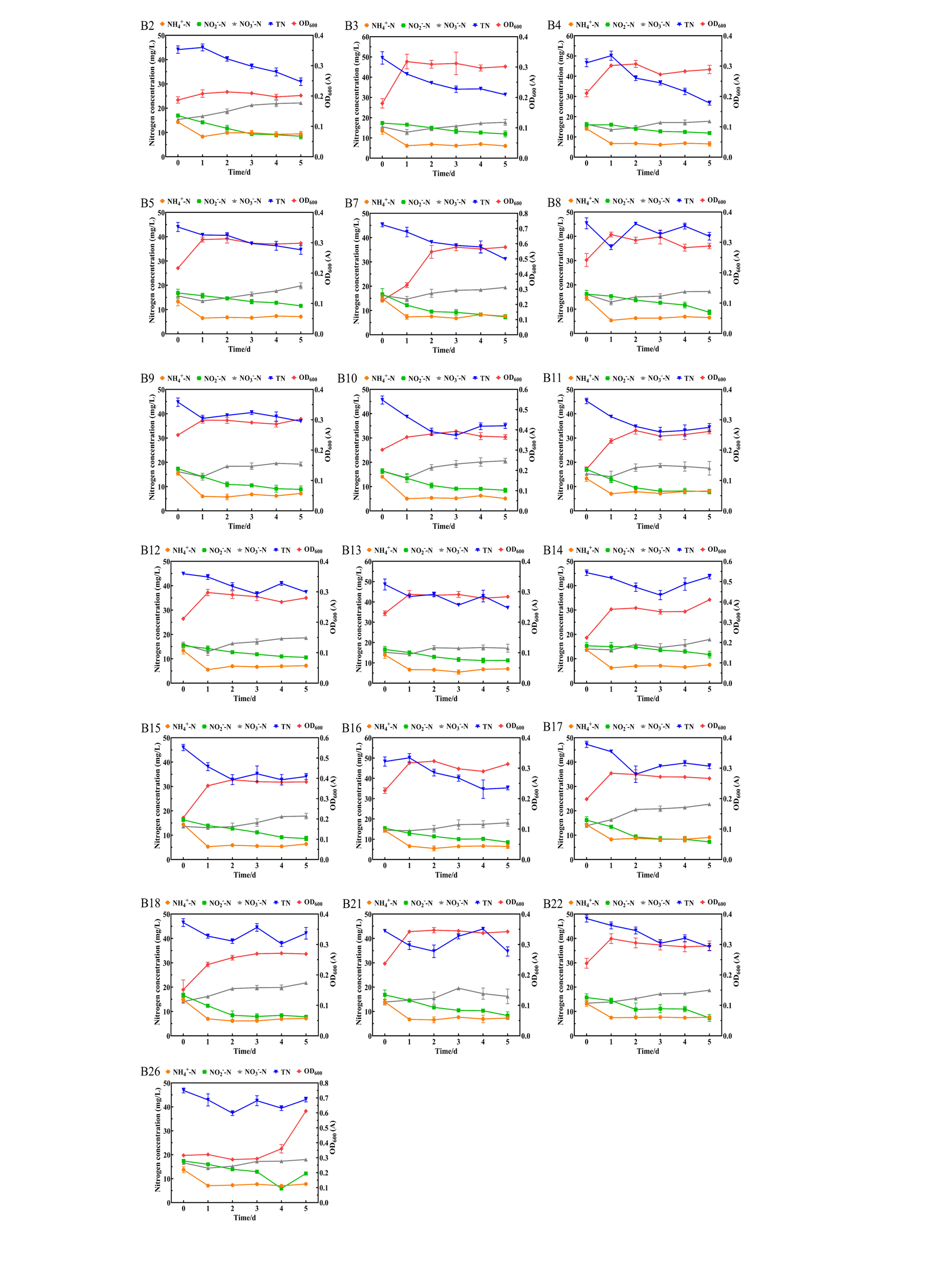

Supplement: S4 Fig — (TIF) [file pone.0319344.s004.tif]
