# Supplementary material for: The nitrogen removal characterization and ecological risk assessment of Bacillus sp. isolated from mariculture systems in China with spatiotemporal difference
Source: PLoS One. 2025 Mar 20;20(3):e0319344. doi: 10.1371/journal.pone.0319344 (PMC11925278; doi:10.1371/journal.pone.0319344)

tetB

used in manuscript

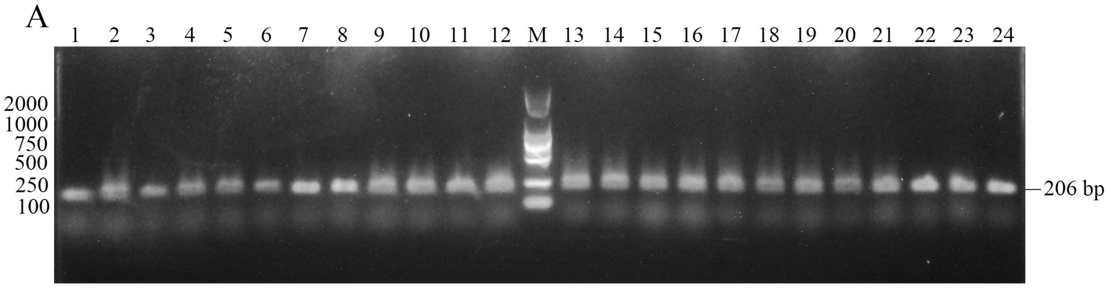

Original gel image

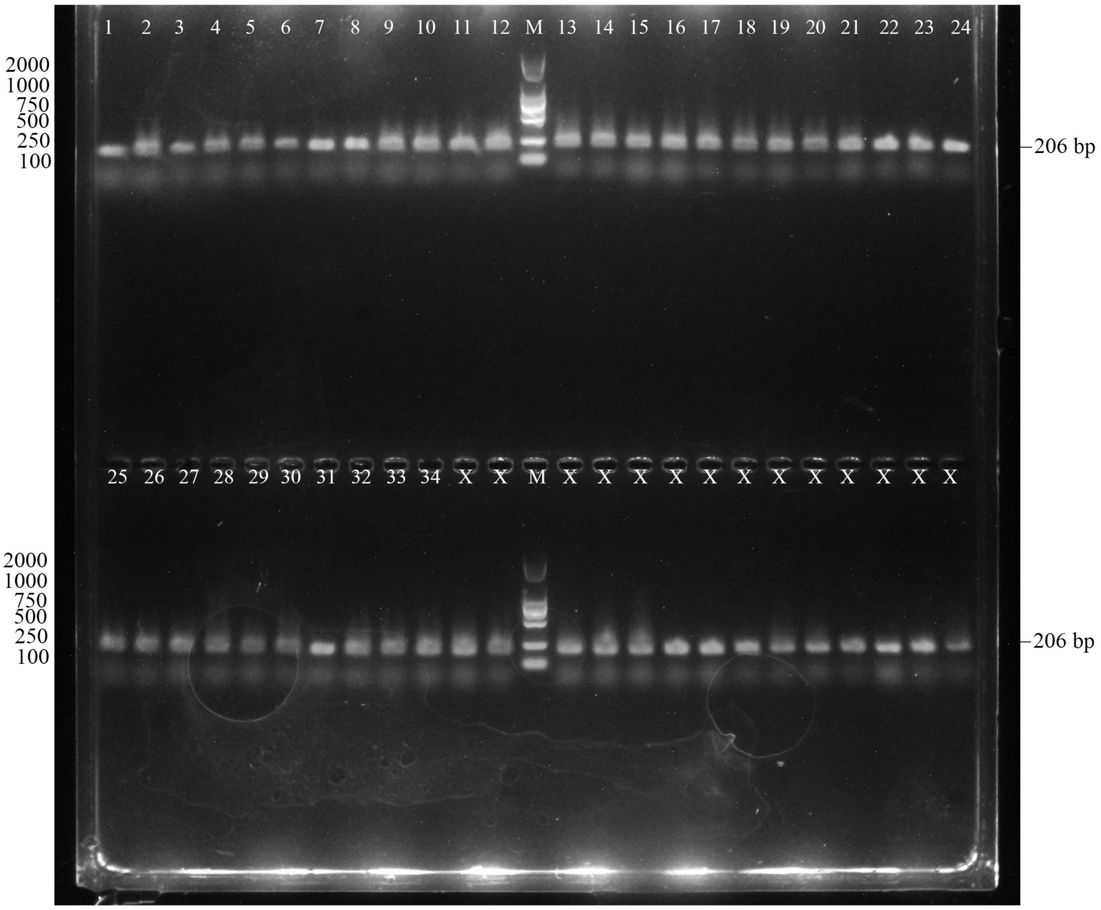

cfr

used in manuscript

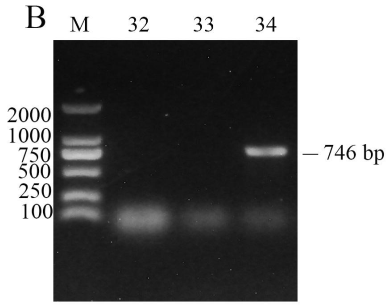

Original gel image

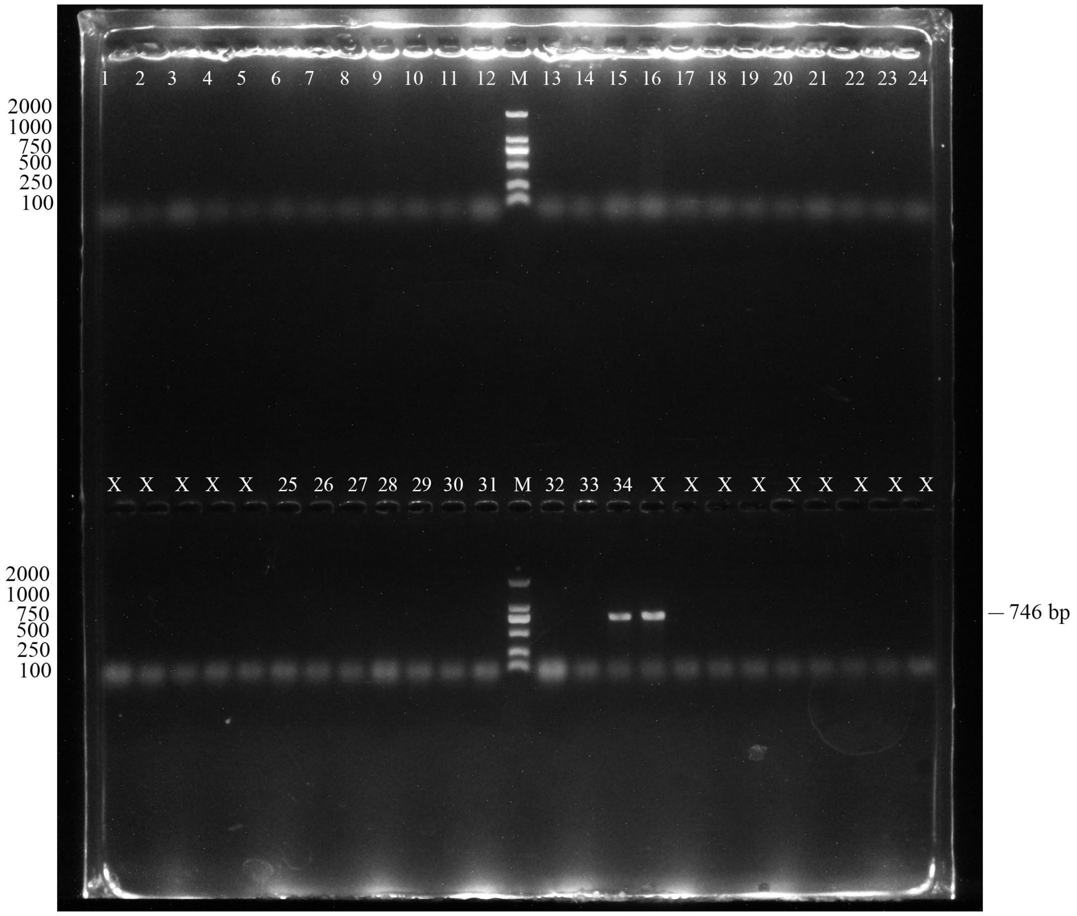

blaTEM

used in manuscript

C

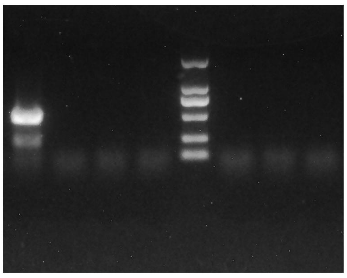

Original gel image

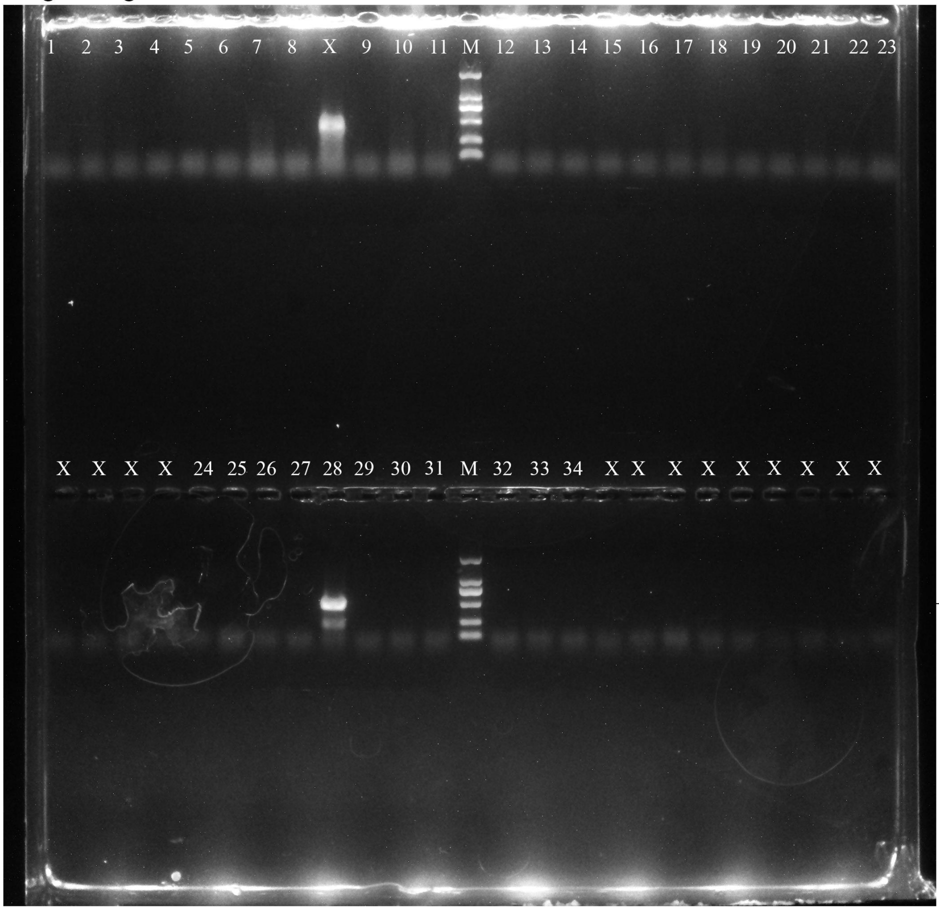

nor used in manuscript

C

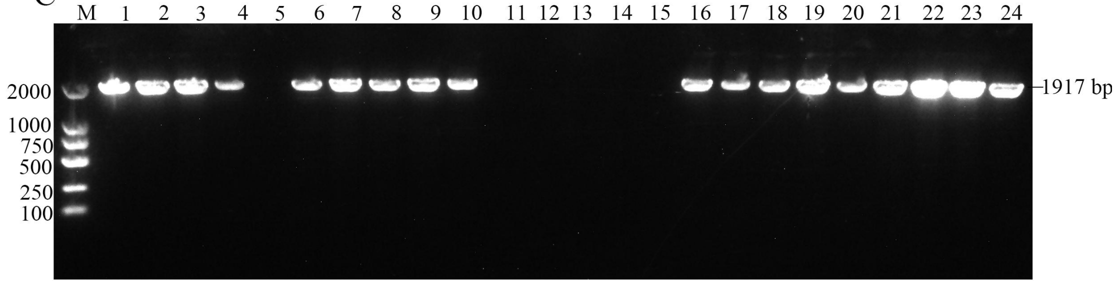

D

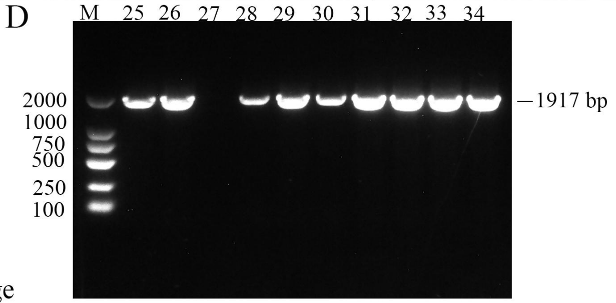

Original gel image

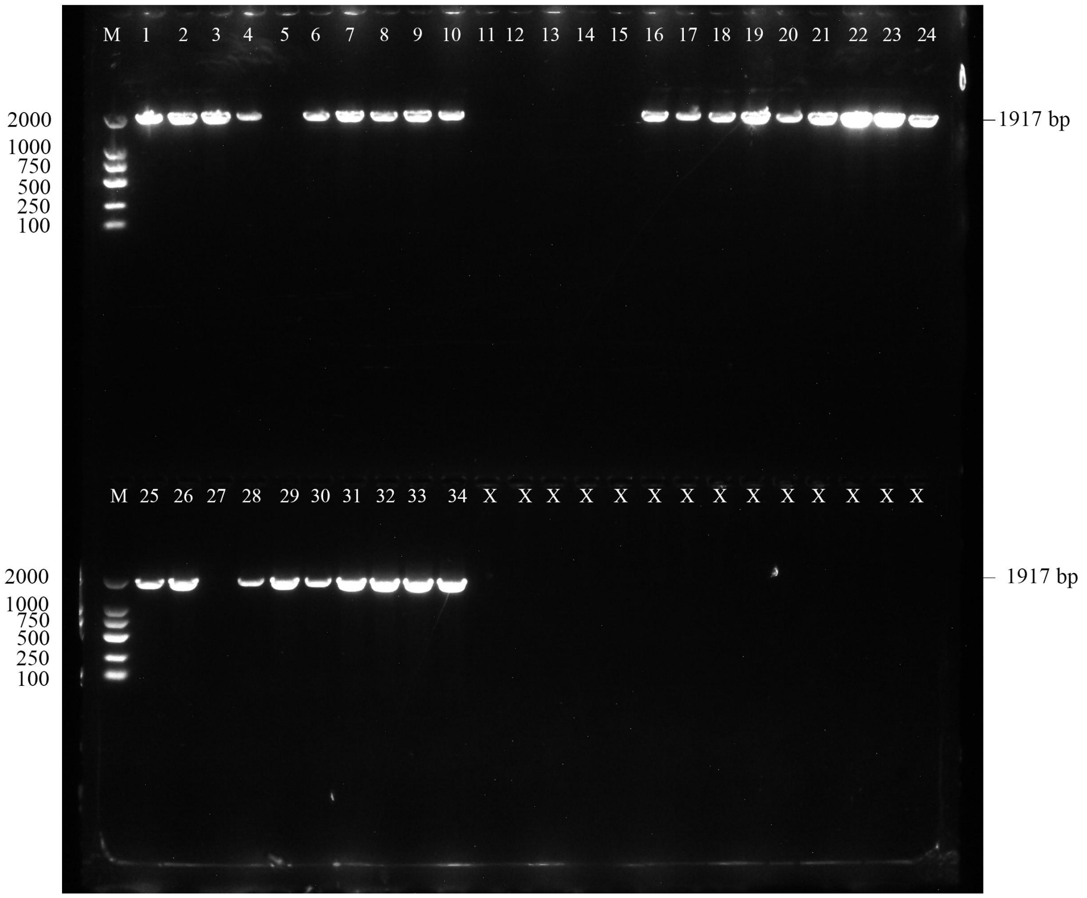

narG used in manuscript

E

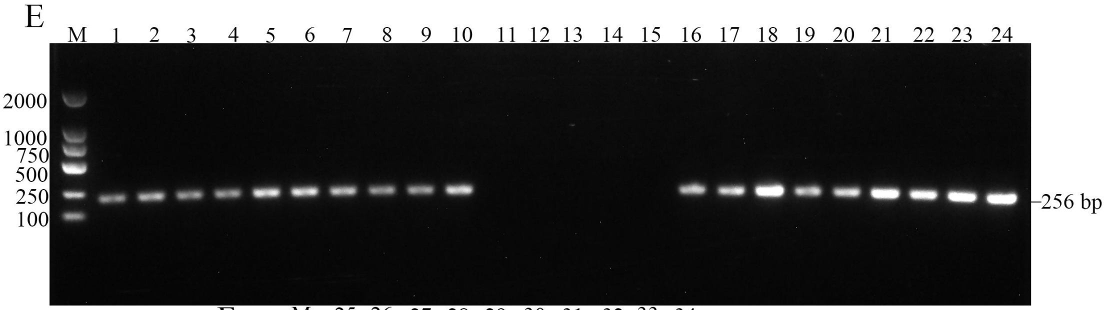

F

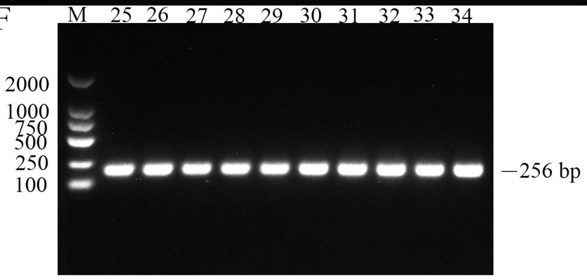

Original gel image

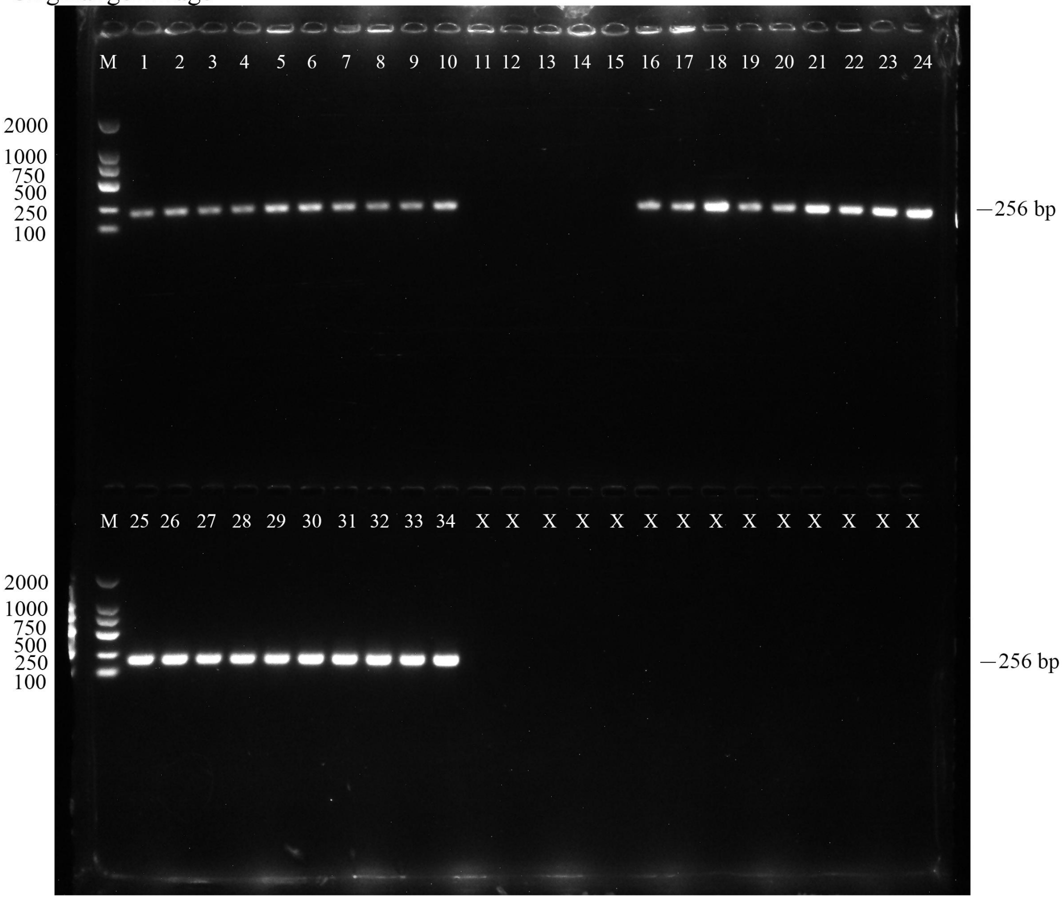

nap used in manuscript

A

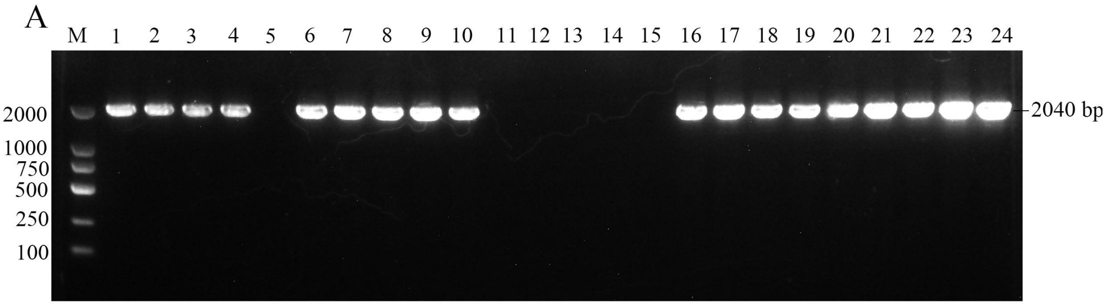

B

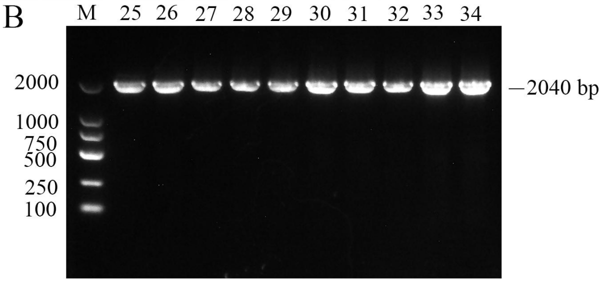

Original gel image

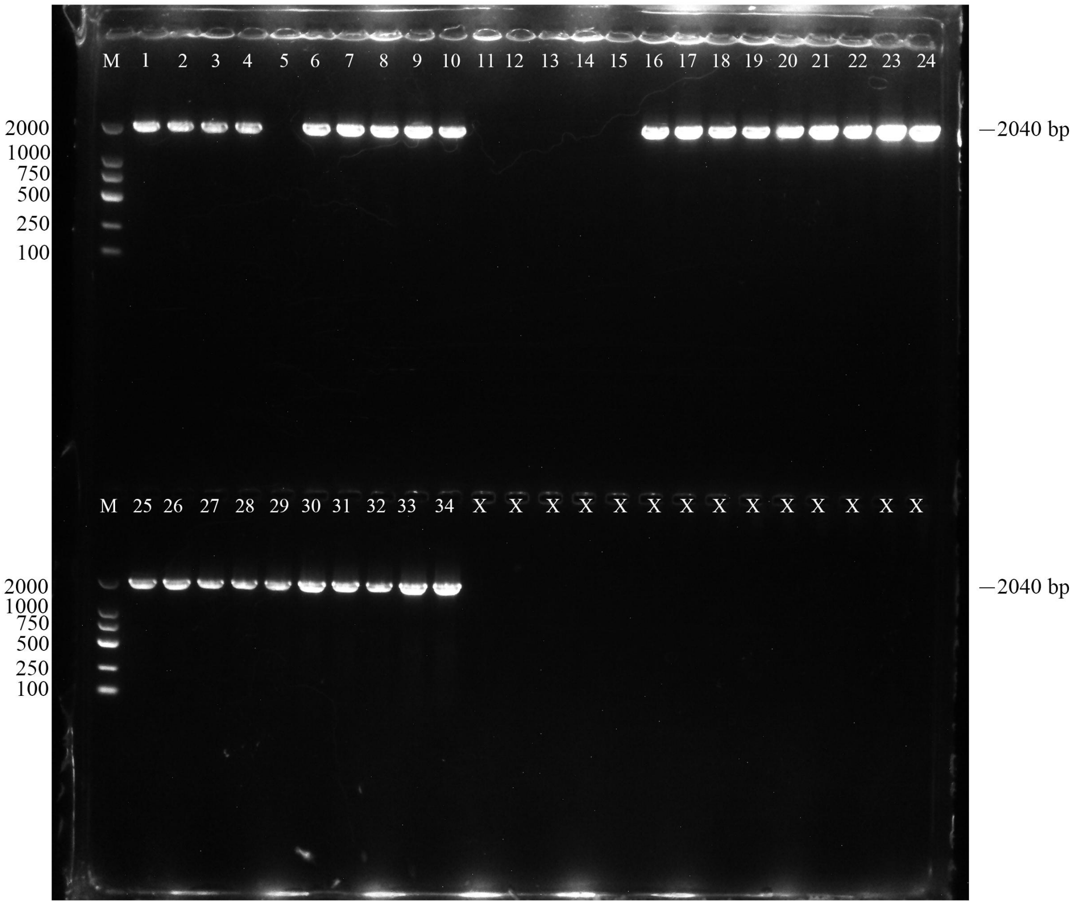

Supplement: S1 Raw Images — (PDF) [file pone.0319344.s005.pdf]
