# Supplementary material for: The nitrogen removal characterization and ecological risk assessment of Bacillus sp. isolated from mariculture systems in China with spatiotemporal difference
Source: PLoS One. 2025 Mar 20;20(3):e0319344. doi: 10.1371/journal.pone.0319344 (PMC11925278; doi:10.1371/journal.pone.0319344)
Supplement: Attachment — Submitted filename: Response to Reviewers.docx [file pone.0319344.s009.docx]

**Dear Editor,**

Thank you and the reviewers for giving us positive evaluation and constructive comments on the manuscript titled “**The nitrogen removal characterization and ecological risk assessment of *Bacillus* sp. isolated from mariculture systems in China with spatiotemporal difference**”. We made a careful response and revision to the manuscript according to the reviewers’ comments. All revisions are listed as follows and marked blue in the revised manuscript. At the same time, We have also updated and modified Figures 1, 3, 4, 5, and 6 according to the journal's requirements, and added the original gel images and related data. We are looking forward to hearing your decision and hope that the revised manuscript will be suitable for publication. Thanks again for your help.

**Reviewer #1:**

**Comment 1:** The abstract is too long and should be further summary and simplification.

**Response:** Thank you very much for your valuable suggestion. Based on your suggestion, we have summarized and simplified the abstract of the paper. Please refer to lines 14-31 in the revised manuscript for specific modifications.

**Comment 2:** The focus and novelty of this paper are not prominent.

**Response:**Thank you for your valuable suggestion. In the revised edition, we further emphasized the focus and novelty of the study. Specifically, we add a discussion of relevant literature in the introduction, and clearly point out how our research can fill the gap of existing research. In addition, in the discussion section, we elaborated the differences and contributions between our findings and existing research, in order to highlight the uniqueness and importance of the research. The revised manuscript were specific modifications.

**Comment 3:** The purpose and signification of the paper in the introduction did not clearly describe and in the last paragraph of the introduction should indicate the content that this paper intends to do, rather than the final experimental results of this paper.

**Response:** We are appreciated for this helpful suggestion. We have made detailed revisions based on your suggestions for the last paragraph of the introduction. Please refer to the revised manuscript for specific modifications.

**Comment 4:** In page 4 line 83-86, this sentence has a grammatical error.

**Response:** Thank you very much for your careful reading and checking. These writing errors have been corrected according to the reviewer’s suggestions. Please refer to lines 90-99 in the revised manuscript for specific modifications.

**Comment 5:** In line 108, line 146-147, line 154, line 162-163, line 185, line 201, line 211, line 263 and line 270-271, there are some issue with those title.

**Response:** Thank you for pointing out the issues with the titles in our paper. We have carefully reviewed the line numbers you mentioned (lines 106, 146, 153, 165-166, 189, 206, 219, 271, and 279-280) and found that there are indeed some inconsistencies in the formatting and wording of the titles. To improve the quality and readability of the paper, we have made the following modifications to these titles.

**line 106:** Preliminary screening of denitrifying *Bacillus* sp.

**line 146:** Amplification of denitrification related genes

**line 153:** Enzyme activity assay

**line 165-166:** Assessment of nitrogen removal capacity

Denitrification performance of *Bacillus* sp. in simulated wastewater

**line 189:** Determination of the virulence of *B. subtilis* B24

**line 206:** Determination of the hemolytic activity of *Bacillus* sp.

**line 219:** Preliminary screening of denitrifying *Bacillus* sp.

**line 271:** Enzyme activity evaluation

**line 279-280:** Assessment of nitrogen removal capacity

The nitrogen removal capacity of *Bacillus* sp. in simulated wastewater

**Comment 6:** The data in the subsequent experimental results of the paper should not appear in the Materials and methods section.
**Response:** Thank you very much for your careful reading and checking. We carefully checked and deleted the experimental results in the "materials and methods" section. Please refer to the revised manuscript for specific modifications.

**Comment 7:** In page 9 line 180-181, the meaning conveyed by this sentence in unclear.

**Response:**Thank you for your valuable guidance. We ensure the clarity of expression in sentences through careful revision. The specific revisions are as following.

The experimental group was inoculated with bacterial suspension once at a ratio of 1% (v/v) (with a concentration of 1×10^9^ CFU/mL per barrel of bacterial suspension). The control group did not add bacteria.

**Comment 8:** The description of the experimental results section is too detailed and should be appropriately simplified. For example, the sentence in line 236-239 can be delete.

**Response:** Thank you for your constructive comment. We have carefully considered your opinion that the experimental results section is too detailed and have made corresponding modifications and deletions. We believe that these modifications will make the article more refined while maintaining the completeness and accuracy of the results. Please refer to the revised manuscript for specific modifications.

**Comment 9:** The content of the discussion section is too scattered and does not highlight the novelty of the paper.

**Response:** Thank you for pointing out that the discussion section is too scattered and does not fully highlight the novelty of the paper. Based on your valuable suggestions, we have made revisions to the discussion section. We have reorganized the content to ensure that each argument closely revolves around the core contribution of our research and highlights the differences between our study and existing literature more clearly. In addition, we also discussed the novelty of our research, emphasized how our findings can fill knowledge gaps, and highlighted the potential impact of our research on the field of aquaculture. We believe that these modifications will make the discussion section more focused and powerful, better showcasing the innovation and importance of our research. Please refer to the revised manuscript for specific modifications.

**Comment 10:** The conclusion section should summarize the results of the paper, rather than describing what the paper has done.
**Response:** We are appreciated for this helpful suggestion. Therefore, we have made corresponding modifications to the conclusion section to ensure a more concise summary of our research results. Please refer to lines 418-429 in the revised manuscript for specific modifications.

**Comment 11:** The nitrogen removal rates of *B. subtilis* B24 was not high, so where is the potential application of this strain? And why not directly screen for efficient degrading Bacillus from natural environments or aquaculture water bodies?

**Response:** Thank you for your valuable comments. Regarding the issue of low nitrogen removal performance and strain sources, our response is as follows. First, concerning the strain source, our strains were isolated from marine aquaculture environments in earlier laboratory experiments, sourced from pathogenic resources in different aquaculture areas, and preserved in the laboratory. For detailed information on the strains, please refer to the materials and methods and Supplementary Table S1. As for the nitrogen removal properties of B24, through the experiments conducted in this study and simulated artificial wastewater experiments, the removal rates of NH_4_^+^-N, NO_2_^-^-N, NO_3_^-^-N, and TN in the simulated wastewater by B24 were 92%, 62%, 68%, and 30%, respectively. This level is considered above average compared to similar studies, indicating that B24 has the potential for efficient nitrogen removal in seawater. However, the effectiveness in the actual seawater application of the mixed wastewater from *Penaeus vannamei* and *Epinephelus* was reduced. The reasons for this difference, apart from the strain itself, also warrant further investigation into environmental and application scenarios. In the future, we will study the cultivation conditions/applicability of the strains and functional gene mining to further clarify their application scenarios and genetic stability, and we will develop and apply them from the perspective of composite microbial agents.

**Reviewer #2:**

**Comment 1:** Line 241, 242. Certain isolates exhibited sensitivity to tetracycline, cephalexin, sulbactam, thiamphenicol and florfenicol, but the genes of *tetB*, *blaTEM* and *cfr* were identified. Please explain Why?

**Response:** Thank you for your valuable questions. We believe that the difference between drug resistance phenotype and drug resistance gene expression may be due to the expression of drug resistance phenotype stimulated by a variety of genetic determinants , or the antimicrobial phenotype is not only mediated by drug resistance genes, but also affected by membrane structure and physiological metabolism. At the same time, bacterial resistance can be divided into metabolic resistance and genetic resistance. Metabolic resistance refers to the strain's own basic metabolic processes, and its tolerance to drugs is non-selective, generally posing no ecological risk. Genetic resistance, on the other hand, occurs because the strain possesses corresponding resistance genes, which not only confer resistance to the strain itself but can also spread in the environment and induce non-resistant bacteria to develop resistance as the strain proliferates and lyses, posing ecological risks. Our goal is not only to understand the metabolic phenotypes of bacterial resistance but also to genetically screen and eliminate strains that pose ecological risks.

**Comment 2:** Line 278-280. Eight isolates maintained a good growth trend, with NH_4_^+^-N removal rates of 80%-92%, NO_2_^-^-N removal rates of 58%-72%, NO_3_^-^-N removal rates of 27%-68%, while the removal rates of TN were only 20%-37%. Please explain “Why”. **Response:**Thank you for your valuable questions. The reasons why these eight strains showed good growth trend but the removal rates of nitrogen-containing compounds were different were explained as follows:

1. NH_4_^+^-N, as a nitrogen source that is easily absorbed and utilized during microbial growth and metabolism, may be largely converted into microbial protein nitrogen through assimilation during the growth and reproduction of strains, or transformed into NO_2_^-^-N/NO_3_^-^-N through nitrification, resulting in a high removal rate.

2. For NO_2_^-^-N, the conversion is mainly achieved through nitrification and denitrification. Although the strains contain nitrite reductase, which can promote the reduction of NO_2_^-^-N, nitrite reductase has biological toxicity that may inhibit the activity of key metabolic enzymes, resulting in a lower removal efficiency compared to NH_4_^+^-N.

3. The removal pathway for NO_3_^-^-N primarily involves its conversion to NO2--N or gaseous nitrogen, which requires complex enzymatic reactions and is influenced by environmental conditions, making removal more challenging and resulting in a relatively low removal rate.

4. Total nitrogen (TN) includes various forms of nitrogen, such as inorganic nitrogen like NH_4_^+^-N, NO_2_^-^-N, and NO_3_^-^-N. Due to the limited removal of NO_2_^-^-N and NO_3_^-^-N, effective removal of NH4+-N alone cannot improve the total nitrogen (TN) removal level. Additionally, the decrease in total nitrogen (TN) is closely related to the final conversion of inorganic nitrogen to gaseous nitrogen discharged from water bodies. Furthermore, a possible insufficient supply of carbon sources may also limit denitrification, which can affect TN removal efficiency.

In conclusion, the differences in the removal efficiency of these strains for different forms of nitrogen may be related to their physiological characteristics, environmental conditions, and carbon source availability. We hope that the above has adequately answered the questions you have raised.

**Comment 3:** In figure 5 and figure 6. It is best to label B1, B6, B19, B20, B23, B24, B25, B27, and control in the figure, respectively.

**Response:** Thank you very much for your valuable suggestion. We strongly agree that clear labelling is essential for the reader's understanding and have updated and revised the labelling of B1, B6, B19, B20, B23, B24, B25, B27 and the control group in Figures 5 and 6 as you suggested, which will make our results clearer and more understandable. Thank you for your attention to the details of the images and your contribution to improving the quality of our report. The specific revisions are as following.

**Fig 5.** **Removal rates of NH_4_^+^-N, NO_2_^-^-N, NO_3_^-^-N, and TN by eight *Bacillus* sp. isolates in inorganic nitrogen degradation ability test medium.** ( B1, B6, B19, B20, B23, B24, B25 and B27) represent the denitrification performance of experimental strains B1, B6, B19, B20, B23, B24, B25 and B27, respectively. (CG) Changes in nitrogen content in the control group.

**Fig 6. Application effect of eight *Bacillus* sp. isolates on nitrogen removal in mariculture wastewater.** ( B1, B6, B19, B20, B23, B24, B25 and B27) represent the nitrogen removal performance of experimental strains B1, B6, B19, B20, B23, B24, B25 and B27, respectively. (CG) variation of nitrogen content in the control group.

**Comment 4:** Line 378-380. NIR enzyme activity could be detected, but *nirS* and *nirK* could not be successfully amplified from the strain. Please explain “Why”.

**Response:** Thank you for your valuable questions.Thank you for your valuable question. Regarding the ability to detect NIR activity in *Bacillus* strains, the *nirS* and *nirK* functional genes were not successfully amplified. This perhaps was explained by the reasons that some new enzymes of unknown function in some of the *Bacillus* species, or some of the denitrification reactions are carried out by chemically-catalyzed processes rather than enzyme-catalyzed, or even the design of the primer sequences is not applicable to these *Bacillus* strains. Therefore, the functional genes and denitrification mechanisms of *Bacillus* need to be further explored at a later stage. Thank you again for your valuable questions.

**Reviewer #3 (Comments for the Author):**

In this manuscript, the authors comprehensively evaluated the nitrogen removal performance and ecological safety of 120 strains of *Bacillus* with spatial and temporal differences. Overall, the data was presented in a scientific way and the results and conclusions are also reasonable. However, some revisions need to be done to improve the manuscript. The authors should take the following suggestions seriously and the grammar errors in the paper should be checked and corrected carefully.
**Response:** Thank you for taking the time to review our manuscript, and thank you for your approval. We have considered all the suggestions and made corresponding corrections. The specific reply is as follows:

**Comment 1:** The paper should be read by a native and some terms should be improved. There are many sentences that need to be rewritten, such as lines 41-42, lines 80-82, lines 90-101, lines 224-225，lines 338-339 and so on.

**Response:** We appreciate your careful review and valuable suggestions.We have thoroughly revised the manuscript for clarity and improved the language throughout. Specifically, we have rewritten the sentences in lines 40-42, 87-89, 90-99, 233-234, and 339-340 to enhance readability and ensure that the terminology used is appropriate.

**Comment 2:** Although 120 strains of Bacillus were used in the study, the details on how these strains adequately represent spatial and temporal differences in mariculture systems of China were lacking. For example, the influence of sample collection standards and culture system types in different regions and time points on strain characteristics was not explained in depth, which may affect the extrapolation of research results.

**Response:** Thank you for your valuable suggestions. In response to the issues raised by the reviewers regarding the unclear background of strain isolation, the authors supplemented the information on the *Bacillus* strains used in the study of denitrification functions by consulting pathogen isolation records and categorizing them based on isolation year, isolation region, host species, or isolation environment. Please refer to S2 Table in the revised manuscript for specific information.

**Comment 3:** The format of the references in lines 48-49 and lines 343-344 is inconsistent with other references.

**Response:** Thank you very much for your careful reading and checking. We have revised the reference format for lines 48-49 and lines 343-344 to align with other references. The specific revisions are as following.

**lines 48-49:** The main sources of nitrogen pollution are NH_4_^+^-N, NO_2_^-^-N, and NO_3_^-^-N [7].

**lines 331-333:** Researchers have evaluated the resistance characteristics of heterotrophic nitrification and aerobic denitrification single strain and its flora to 22 antibiotics such as macrolides, tetracyclines, mycins, and sulfonamides [13].

**Comment 4:** In the process of discussion, some contents were not logical enough. For example, when describing the safety of *Bacillus* as an aquatic probiotic, there was a direct jump from hemolysis test to antibiotic sensitivity, and there was a lack of transitional analysis in the middle. The logical correlation between different results should be strengthened to make the discussion more coherent and smooth. **Response:** We greatly appreciate your valuable suggestions on the logical relationship discussed in the article. We have revised the discussion section to include a detailed analysis of the logical relationship between hemolysis testing and antibiotic sensitivity. We have added transitional sentences and explanations to ensure that readers can clearly understand the connection between the two, thereby enhancing the coherence and fluency of the discussion.

**Comment 5:** The marking of some Figures is not perfect enough. The strain number represented by each curve in Figure 5 is not marked directly, so it needs to be found in the text, which affects the independence and readability of the Figure. It should be marked clearly in the Figure, so that readers can understand the meaning of the Figure without referring to the text.
**Response:** Thank you very much for your valuable suggestion. We have directly labeled the strain numbers represented by each curve in Fig 5 to improve the independence and readability of the graph, so that readers can understand the meaning of the graph without referring to the text. The specific revisions are as following.

**Fig 5.** **Removal rates of NH_4_^+^-N, NO_2_^-^-N, NO_3_^-^-N, and TN by eight *Bacillus* sp. isolates in inorganic nitrogen degradation ability test medium.** ( B1, B6, B19, B20, B23, B24, B25 and B27) represent the denitrification performance of experimental strains B1, B6, B19, B20, B23, B24, B25 and B27, respectively. (CG) Changes in nitrogen content in the control group.

**Comment 6:** In the part of hemolysis analysis, the description of the experimental operation is clear, but the specific criteria for determining the type of hemolysis (strong, weak, and non-hemolysis) are not clearly defined. For example, the corresponding relationship between the size of the hemolysis circle and the type of hemolysis may lead to the difference in judgment between different experimenters, and detailed criteria should be added.

**Response:** Thank you for your valuable suggestions. To avoid confusing readers regarding the understanding of hemolytic results, we have classified the hemolysis test results into two standards: hemolytic and non-hemolytic. In the revised version, we have changed the detailed standards for hemolytic types, including the specific correspondence between hemolytic zone size and hemolytic type. These standards include specific diameter ranges to distinguish between hemolytic and non-hemolytic types. The specific modifications are as follows.

It was preliminarily inferred that these 55 *Bacillus* sp. isolates posed no pathogenic risk. The types of haemolysis were classified as haemolytic and non-haemolytic based on the size of the haemolytic circle diameter. The diameter of the haemolytic circle is greater than 0 mm for haemolytic and no haemolytic circle for non-haemolytic. Fig 1 shows a schematic diagram of haemolytic and non-haemolytic of *Bacillus* sp. isolates.

**Comment 7:** It is found that strains are sensitive to some antibiotics but carry corresponding resistance genes. Can we explore the molecular mechanism, such as gene regulation mechanism and detection of drug-resistance gene expression level, to explain this contradictory phenomenon.

**Response:** Thank you very much for your valuable suggestions. We very much agree with your idea, and we will also explore this paradoxical phenomenon in the follow-up experiments. Meanwhile, we believe that the difference between the resistance phenotype and the expression of resistance genes may be due to the fact that the expression of the resistance phenotype is stimulated by a variety of genetic determinants, or that the antibacterial phenotype is not only mediated by resistance genes, but also influenced by membrane structure and physiological metabolism. In addition, bacterial resistance can be categorised into metabolic and genetic resistance. Metabolic resistance refers to the basic metabolic process of the strain itself, which is non-selective in its tolerance to drugs and is generally not ecologically harmful. Genetic resistance, on the other hand, is due to strains having corresponding resistance genes that not only confer resistance to the strain itself, but can also be transmitted in the environment and pose an ecological risk by inducing resistance in non-resistant bacteria as the strain proliferates and lyses. Our goal is not only to understand the metabolic phenotype of bacterial resistance, but also to genetically screen and eliminate ecologically harmful strains.

**Comment 8:** The introduction of various media components is too lengthy, and it can be focused on the key components and effects directly related to experimental screening or detection, simplifying the description of conventional components (such as glucose, sodium chloride, etc.).

**Response:** Thank you very much for your valuable suggestions. We have simplified the presentation of the media components and included them as supplementary information. Ensure that the focus is on media and their functions that are directly relevant to experimental screening and assays to improve the conciseness and readability of the article. Please refer to line 90-99 and S1 Table in the revised manuscript for specific modifications.

**Comment 9:** When discussing the safety and drug resistance characteristics of *Bacillus* as a probiotic, literatures such as [12], [32] and [33] were cited, but only the results of previous studies were briefly mentioned, without in-depth analysis of the specific differences and connections between these literatures and the characteristics of strains and experimental results in this study. For example, literature [12] describes the nitrifying and denitrifying characteristics of a new strain of *Bacillus*. Although the denitrifying function of *Bacillus* is involved in this study, the characteristics of the strain in this literature were not compared with the strains screened in this study in terms of drug resistance and ecological safety, which makes the reference seem superficial. The supporting role of cited literature for in-depth discussion of research results is not fully utilized.

**Response:** Thank you for your valuable advice. We have updated these references in the revised manuscript with more in-depth analyses of the differences and connections with our findings, emphasising the importance of our study, strengthening the logic of the exposition, and ensuring that the reader can clearly understand the relationships and differences between the various literatures. Please refer to line 348-351 in the revised manuscript for specific modifications.

**Comment 10:** For some key experimental operations and detection methods (such as enzyme activity detection methods), there is no reference to provide method basis or comparison with existing research methods, which makes the innovation and reliability of experimental methods lack sufficient literature support, and it is difficult to judge the status and applicability of these methods in this field of research.

**Response:** Thank you for your valuable suggestion. We have added detailed descriptions of key experimental operations and detection methods in the revised manuscript, emphasizing the reliability of our method to enhance readers' understanding of our research. The specific modifications are as follows.

Inoculate the activated 27 *Bacillus* sp. into TSB liquid medium, culture for 16 h, and collect the bacterial solution by centrifugation (6000 rpm, 4 ℃, 10 min). Crush the bacterial cells with ultrasound (300W, 20 min) and collect the supernatant as the crude enzyme solution. The activity of HAO, NAR, NIR, and AMO in the crude enzyme solution was detected by using the hydroxylamine oxidoreductase (HAO) kit, nitrate reductase (NAR) kit, nitrite reductase (NIR) kit, and ammonia monooxygenase (AMO) kit (mlbio, Shanghai, China). The protein content was detected using the BCA method (mlbio, Shanghai, China). Each group was repeated three times, and no enzyme extract was added to the control group.

**Comment 11:** The expression format of Figure numbers should be unified to enhance the standardization of article format. For example, when referring to Figure 1 in the manuscript, it was expressed as "Fig 1", while when referring to other figures, some of them were expressed in different forms, such as "Figure 1" and "Fig. 1".

**Response:** Thank you for your valuable suggestions. We have revised the manuscript and standardised the format for expressing figure numbers. A uniform format is now used for all figure references to ensure the standardisation of the article format. Please refer to the revised manuscript for details of the revisions. Please refer to the revised manuscript for specific modifications.

**Comment 12:** In the description of experimental results and discussion, the technical terms are not consistent enough for the expression of some similar concepts or indicators. For example, when describing nitrogen compounds, "ammonium", "ammonia nitrogen", "NH_4_^+^-N" and other expressions are used alternately, although the meaning is similar, but in the same article should try to uniform terminology.

**Response:** Thank you for your constructive comment. We have carefully reviewed the terminology used in the article and revised the relevant sections to ensure that the term "NH_4_^+^- N" is uniformly used when describing nitrogen compounds, avoiding the mixing of expressions such as "ammonia nitrogen" and "ammonium".

**Comment 13:** Although repeated experiments on data accuracy and reliability were mentioned in the paper, the statistical analysis method used was not clearly explained in the comparison and analysis of multiple experimental results. For example, when comparing data such as differences in nitrogen removal rates and drug resistance among different strains, it was not mentioned what statistical tests (such as T-test, ANOVA, etc.) were used and what significance level was set.

**Response:** Thank you for your valuable suggestions. We apologise for the confusion caused by the incorrect description we used when comparing the denitrification rate and resistance of the strains. When analysing the denitrification efficiency and resistance of the strains, we performed three replications for each group of strains and the statistics are expressed as mean ± standard deviation and the results were not subjected to significance analysis, but simply compared the size of the data. For statements in the manuscript that confused you, we have updated them in the revised manuscript.
