# Supplementary material for: The nitrogen removal characterization and ecological risk assessment of Bacillus sp. isolated from mariculture systems in China with spatiotemporal difference
Source: PLoS One. 2025 Mar 20;20(3):e0319344. doi: 10.1371/journal.pone.0319344 (PMC11925278; doi:10.1371/journal.pone.0319344)
Supplement: Attachment — Submitted filename: Comments on PONE-D-24-56326.docx [file pone.0319344.s007.docx]

In this manuscript, the authors comprehensively evaluated the nitrogen removal performance and ecological safety of 120 strains of *Bacillus* with spatial and temporal differences. Overall, the data was presented in a scientific way and the results and conclusions are also reasonable. However, some revisions need to be done to improve the manuscript. The authors should take the following suggestions seriously and the grammar errors in the paper should be checked and corrected carefully.

1. The paper should be read by a native and some terms should be improved. There are many sentences that need to be rewritten, such as lines 41-42, lines 80-82, lines 90-101, lines 224-225，lines 338-339 and so on.
2. Although 120 strains of *Bacillus* were used in the study, the details on how these strains adequately represent spatial and temporal differences in mariculture systems of China were lacking. For example, the influence of sample collection standards and culture system types in different regions and time points on strain characteristics was not explained in depth, which may affect the extrapolation of research results.
3. The format of the references in lines 48-49 and lines 343-344 is inconsistent with other references.
4. In the process of discussion, some contents were not logical enough. For example, when describing the safety of *Bacillus* as an aquatic probiotic, there was a direct jump from hemolysis test to antibiotic sensitivity, and there was a lack of transitional analysis in the middle. The logical correlation between different results should be strengthened to make the discussion more coherent and smooth.
5. The marking of some Figures is not perfect enough. The strain number represented by each curve in Figure 5 is not marked directly, so it needs to be found in the text, which affects the independence and readability of the Figure. It should be marked clearly in the Figure, so that readers can understand the meaning of the Figure without referring to the text.
6. In the part of hemolysis analysis, the description of the experimental operation is clear, but the specific criteria for determining the type of hemolysis (strong, weak, and non-hemolysis) are not clearly defined. For example, the corresponding relationship between the size of the hemolysis circle and the type of hemolysis may lead to the difference in judgment between different experimenters, and detailed criteria should be added.
7. It is found that strains are sensitive to some antibiotics but carry corresponding resistance genes. Can we explore the molecular mechanism, such as gene regulation mechanism and detection of drug-resistance gene expression level, to explain this contradictory phenomenon.
8. The introduction of various media components is too lengthy, and it can be focused on the key components and effects directly related to experimental screening or detection, simplifying the description of conventional components (such as glucose, sodium chloride, etc.).
9. When discussing the safety and drug resistance characteristics of *Bacillus* as a probiotic, literatures such as [12], [32] and [33] were cited, but only the results of previous studies were briefly mentioned, without in-depth analysis of the specific differences and connections between these literatures and the characteristics of strains and experimental results in this study. For example, literature [12] describes the nitrifying and denitrifying characteristics of a new strain of *Bacillus*. Although the denitrifying function of *Bacillus* is involved in this study, the characteristics of the strain in this literature were not compared with the strains screened in this study in terms of drug resistance and ecological safety, which makes the reference seem superficial. The supporting role of cited literature for in-depth discussion of research results is not fully utilized.
10. For some key experimental operations and detection methods (such as enzyme activity detection methods), there is no reference to provide method basis or comparison with existing research methods, which makes the innovation and reliability of experimental methods lack sufficient literature support, and it is difficult to judge the status and applicability of these methods in this field of research.
11. The expression format of Figure numbers should be unified to enhance the standardization of article format. For example, when referring to Figure 1 in the manuscript, it was expressed as "Fig 1", while when referring to other figures, some of them were expressed in different forms, such as "Figure 1" and "Fig. 1".
12. In the description of experimental results and discussion, the technical terms are not consistent enough for the expression of some similar concepts or indicators. For example, when describing nitrogen compounds, "ammonium", "ammonia nitrogen", "NH_4_^+^-N" and other expressions are used alternately, although the meaning is similar, but in the same article should try to uniform terminology.
13. Although repeated experiments on data accuracy and reliability were mentioned in the paper, the statistical analysis method used was not clearly explained in the comparison and analysis of multiple experimental results. For example, when comparing data such as differences in nitrogen removal rates and drug resistance among different strains, it was not mentioned what statistical tests (such as T-test, ANOVA, etc.) were used and what significance level was set.
